# Linkage analysis, GWAS, transcriptome analysis to identify candidate genes for rice seedlings in response to high temperature stress

**DOI:** 10.1186/s12870-021-02857-2

**Published:** 2021-02-09

**Authors:** Zhaoran Wei, Qiaoling Yuan, Hai Lin, Xiaoxia Li, Chao Zhang, Hongsheng Gao, Bin Zhang, Huiying He, Tianjiao Liu, Zhang Jie, Xu Gao, Shandang Shi, Bo Wang, Zhenyu Gao, Lingrang Kong, Qian Qian, Lianguang Shang

**Affiliations:** 1 Shenzhen Branch, Guangdong Laboratory for Lingnan Modern Agriculture, Genome Analysis Laboratory of the Ministry of Agriculture, Agricultural Genomics Institute at Shenzhen, Chinese Academy of Agricultural Sciences, Shenzhen, China; 2 State Key Laboratory of Crop Biology, College of Agriculture, Shandong Agricultural University, Tai’an, 271018 Shandong China; 3 State Key Laboratory of Rice Biology, China National Rice Research Institute, Chinese Academy of Agricultural Sciences, Hangzhou, 310006 China

**Keywords:** Linkage analysis, GWAS, Transcriptome analysis, Rice seedling, High-temperature-mediated growth response

## Abstract

**Background:**

Rice plants suffer from the rising temperature which is becoming more and more prominent. Mining heat-resistant genes and applying them to rice breeding is a feasible and effective way to solve the problem.

**Result:**

Three main biomass traits, including shoot length, dry weight, and fresh weight, changed after abnormally high-temperature treatment in the rice seedling stage of a recombinant inbred lines and the natural *indica* germplasm population. Based on a comparison of the results of linkage analysis and genome-wide association analysis, two loci with lengths of 57 kb and 69 kb in *qDW7* and *qFW6*, respectively, were associated with the rice response to abnormally high temperatures at the seedling stage. Meanwhile, based on integrated transcriptome analysis, some genes are considered as important candidate genes. Combining with known genes and analysis of homologous genes, it was found that there are eight genes in candidate intervals that need to be focused on in subsequent research.

**Conclusions:**

The results indicated several relevant loci, which would help researchers to further discover beneficial heat-resistant genes that can be applied to rice heat-resistant breeding.

**Supplementary Information:**

The online version contains supplementary material available at 10.1186/s12870-021-02857-2.

## Background

Rice (*Oryza sativa* L*.*) is the main food crop worldwide, especially in Asian countries*.* As an Asian country, China has a population of 1.4 billion, and 2/3 of the people’s main food is rice. At the same time, China is the world’s largest rice producer whose rice production ranks first in the world [1]. But, as the global industrialization process accelerates, greenhouse gases, such as carbon dioxide (CO_2_) and methane (CH_4_), have caused the global temperature to rise continually*.* The rising high temperature is affecting the rice yield worldwide*.* Even if some rice varieties from tropical regions have formed ecological characteristics adapted to high-temperature environments, their yields will also be affected once the temperature exceeds their appropriate temperature [2]. The ambient temperature exceeding the appropriate temperature during rice growth and development would lead to yield reduction [3]. Some high-temperature sensitive varieties may have a decrease in the yield of more than 10% for every 1 °C increase [4]. Early seedling growth is important for plant morphogenesis, but high-temperature stress during this period is unfavorable for plant growth and development [5]. When rice seedlings experience high temperatures above 35 °C, the protein type and content of leaves change considerably [6, 7], and the shoot length, dry weight, and other traits are negatively affected [8].

To figure out the mechanism of rice responding to high-temperature stress, some research groups have used mapping populations to explore the effects of high-temperature stress on rice and obtained heat-tolerant genes or the quantitative trait locus (QTLs) at different physiological stages [5, 6, 9–11]. Thus, linkage analysis has been proved to be a feasible method for mining heat-resistant genes in rice. However, as the number of inbred line populations used in linkage analysis is limited, the QTL results have a wide range [12], and the detected QTL results are difficult to be used directly in breeding, which requires time-consuming fine mapping [2]. Association analysis using natural populations can effectively circumvent the problem of linkage analysis*.* So far, there are few reports on using natural populations to mine beneficial loci in response to heat stress during rice vegetative growth*.*

Genome-wide association analysis (GWAS) has been used to identify causal loci for important agronomic traits, such as grain length [13, 14] and the 1000-grain weight [13]. Previously, some key single nucleotide polymorphisms (SNPs) on chromosome 4 affecting floret fertility were detected under high-temperature conditions using GWAS population [15]. However, the influences of the population genetic structure and allele frequency were easily overlooked in GWAS, which increased the probability of false positives in linkage disequilibrium (LD) mapping results [16]. Combining the results from linkage analysis and correlation analysis has facilitated the attempt to explore quantitative agronomic traits*.* It has been confirmed that combining these methods can further improve the efficiency and accuracy of QTL calling [17] and can effectively narrow the intervals of major QTLs in rice [18]. In this study, a natural rice population and the recombinant inbred lines (RIL) population derived from the 93–11 × PA64s were used to compare the differences in seedling traits with or without heat stress treatment, and then linkage analysis, association analysis and ribonucleic acid sequencing (RNA-seq) analysis were conducted*.* QTLs related to heat stress in the rice seedling stage were identified, which provided a basis for later fine mapping and gene editing in heat-resistant molecular-assisted selection breeding*.*

## Results

### Phenotypes of RIL and GWAS population

**Fig. 1:**
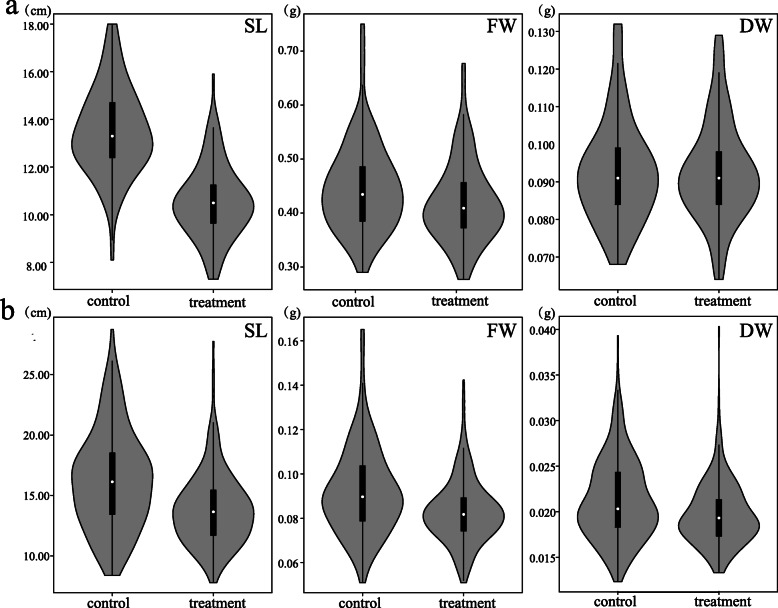
The phenotypic characters of RIL and the natural population. **a** Phenotypic characters of the RIL used for linkage analysis. **b** Phenotypic characters of the natural rice population used for association analysis. SL, FW, DW indicate shoot length, fresh weight, and dry weight of the seedlings

To detect the influence of heat treatment on rice seedlings, the phenotypic characters of RIL and the natural rice population with or without heat treatment were statistically analyzed (Fig*.* 1 and S1)*.* The average and extreme values of the shoot length (SL), dry weight (DW), and fresh weight (FW) of RIL and natural population decreased after heat stress treatment, which confirmed that heat stress had a negative impact on rice growth*.* The measured values between the RIL control and the treatment groups were different, and the measured values were higher than the average value of the corresponding parents, which suggested the existence of super-parent separation of biomass traits (Table S1)*.* The measured traits of the natural rice population and the RIL were tested to determine whether they followed a normal distribution (Fig. S2 and S3)*.* It showed that shoot length, dry weight, and fresh weight were basically in accordance with a normal distribution in both RIL and GWAS population*.* According to the traits data, it was determined that the biomass traits of rice seedlings in the experiment are quantitative traits controlled by typical poly-genes (Table S1)*.* Biomass traits of the control and treatment groups in RIL were significantly lower than those in the parental groups*.* However, the survival rate (SR) of the RIL was higher compared to the parents (Fig*.* 1, Table S1)*,* which suggested that the RIL had pyramided more heat-tolerant genes from the parental materials during the recombination process*.* The difference in biomass traits between the control and treatment groups of GWAS population was less than that of RIL, while the SR of GWAS population was significant higher than that of RIL (Fig*.* 1, Table S1)*.* These results inferred that there were more natural heat-tolerant genes and genetic diversity in the natural population*.* Exploring the heat-resistant QTLs of rice seedlings provides a possibility for comparison of control and post-treatment QTLs mapping to obtain QTLs for heat-resistant effects.

**Fig. 2:**
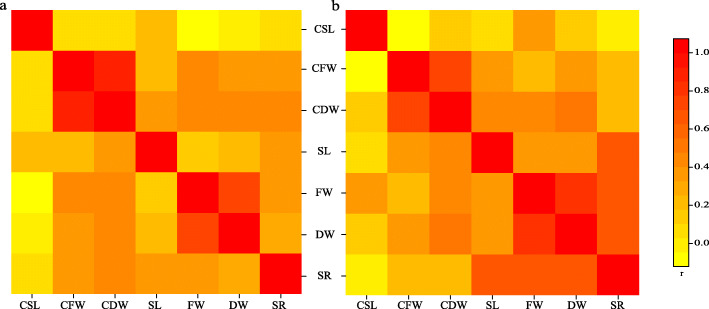
Correlation analysis among biomass traits of RIL and the natural population. **a** Recombinant inbred lines. **b** Natural rice population. The color indicates the correlation coefficient. CSL, CFW, and CDW indicate shoot length, fresh weight, and dry weight of the seedlings in control groups, respectively. SL, FW, and DW indicate the respective shoot length, fresh weight, and dry weight of the seedling in treatment groups, respectively. SR indicates survival rate of the treatment

The correlations among biomass traits of RIL and GWAS population were analyzed separately (Fig. 2). The results showed that the correlations of the biomass traits between the two groups were different. Among them, the strongest correlation was found between dry weight and fresh weight of the GWAS treatment group, and the correlation coefficient was 0.91. In the RIL population, there was also a strong correlation between dry weight and fresh weight of the RIL treatment group, with a correlation coefficient of 0.75. These results suggested that there should be genes in rice seedlings that affected both dry and fresh weight of rice seedlings simultaneously under high temperature stress and responded to high temperature stress in this way. Previous studies have confirmed that seedling biomass traits can reflect rice seedling vigor [19]. But in this study, although the effect of high temperature stress on rice seedlings could be directly reflected by biomass traits, correlation analysis showed that there was a very weak correlation between survival rate traits that directly reflected the vigor of rice seedlings and the biomass of rice seedlings. This indicates that biomass traits are not the unique manifestation in response to high temperature stress in rice and that survival rates and biomass traits are regulated by different genes.

### QTL mapping of RIL

Using the high-density map of RIL in 2013 [20], QTL mapping of the RIL with or without heat stress treatment was carried out. In total, 20 QTLs, distributed on the chromosomes 2, 3, 4, 6, 7, 9,11, and 12, were detected (Table 1). The detection of multiple QTLs for the same trait indicates that the biomass trait is indeed a quantitative trait. There are several known genes related to plant heat tolerance in the above QTL intervals, including *GS8* [21] and *abl1* [22]. All of these known genes confirmed the accuracy of the linkage analysis. The results showed that *qDW11*, a locus for dry weight trait in the treatment group, had the highest logarithm of odds (LOD) value (6.72). Meanwhile, we found *qDW11* and another loci *qFW11.1* for fresh weight in the treatment group are two identical intervals, which is also verified by the correlation analysis results (Fig. 2). In the analysis of the dry and fresh weight of the control group, the same set of QTLs (*qCDW11* and *qCFW11*) are also identified on chromosome 11. However, the same interval for dry and fresh weight detected in the control group was inconsistent with that in the treatment group, indicating that the QTLs identified in the treatment group responded to high temperature. Therefore, we speculate that *qDW11* and *qFW11.1* contain genes that respond to high temperature stress, which are not expressed under normal growth conditions, and we will focus on this candidate interval later.

**Table 1:** QTLs for various traits during the seedling stage

Trait	QTL	Chromosome	LOD	Genetic Distance (cM)	Known Gene
CDW	*qCDW2.1*	2	2*.*88	98*.*2–101*.*3	
	*qCDW2.2*	2	3*.*65	105*.*8–107*.*8	
	*qCDW2.3*	2	3*.*30	142*.*5–145*.*3	
	*qCDW11*	11	4*.*01	8*.*1–9*.*4	
CFW	*qCFW6.1*	6	3*.*09	58*.*3–60*.*1	
	*qCFW6.2*	6	2*.*92	64*.*2–65*.*7	
	*qCFW11*	11	3*.*59	7*.*8–9*.*4	
CSL	*qCSL4.1*	4	2*.*57	74*.*4–78*.*3	
	*qCSL4.2*	4	3*.*81	84*.*2–85*.*8	
	*qCSL9*	9	5*.*15	27*.*3–29*.*1	
DW	*qDW3*	3	3*.*82	11*.*0–14*.*3	*GS8* [21]
	*qDW7.1*	7	3*.*68	28*.*0–29*.*0	
	*qDW11*	11	6*.*72	60*.*5–63*.*5	
FW	*qFW6*	6	2*.*77	19*.*1–22*.*0	*abl1* [22]
	*qFW11.1*	11	5*.*01	60*.*5–64.0	
	*qFW11.2*	11	2*.*53	66*.*1–70*.*4	
SL	*qSL12.1*	12	4*.*80	9*.*7–11*.*5	
	*qSL12.2*	12	2*.*82	21*.*9–25*.*9	
SR	*qSR3.1*	3	2*.*96	122*.*3–125*.*1	
	*qSR3.2*	9	3*.*54	2.0–7*.*5	

CSL, CFW, and CDW indicate shoot length, fresh weight, and dry weight of the seedlings in control groups, respectively*.* SL, FW, and DW indicate shoot length, fresh weight, and dry weight of the seedling in treatment groups, respectively*.* SR indicates the survival rate

### GWAS of heat-related traits in the seedling stage

**Fig. 3:**
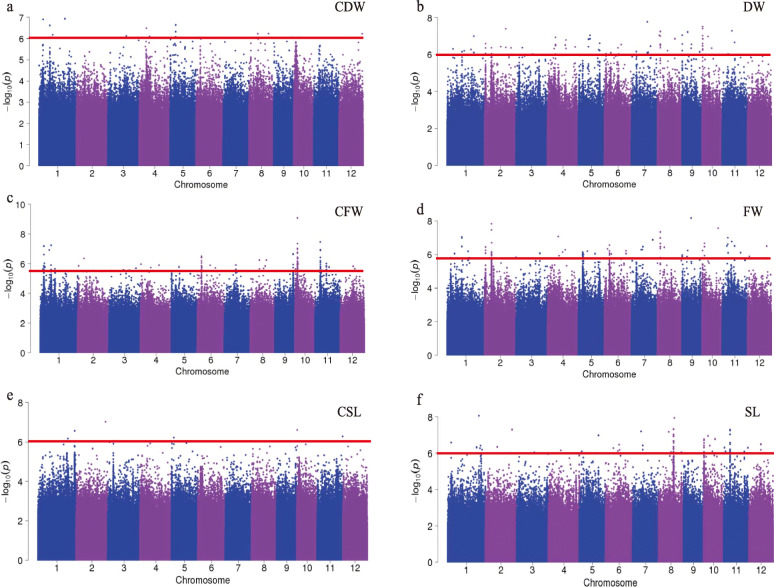
Genome-wide association analysis of biomass traits under heat stress. Manhattan plots of **a** Dry weight of control groups. **b** Dry weight of treatment groups. **c** Fresh weight of control groups. **d** Fresh weight of treatment groups. **e** Shoot length of control groups. **f** Shoot length of treatment groups. CSL, CFW, and CDW indicate shoot length, fresh weight, and dry weight of the seedlings in control groups, respectively. SL, FW, and DW indicate the respective shoot length, fresh weight, and dry weight of the seedling in treatment groups, respectively

A genome-wide association analysis of 255 natural rice lines (Table S3) was performed using the existing genome-wide coverage of 14,779,691 SNPs data from 3 k database [23]. It is generally considered that LD is between 100 kb and 200 kb in rice [18]. In this study, the SNP threshold for the significantly associated sites is *P*-value < 10^− 6^ and the significant SNPs sites within the 100 kb interval are considered candidate loci (Table S3)*.* There were a large number of different significant loci in the heat treatment and control groups, which suggested that the high temperature in the seedling stage might affect the growth of the natural population through these loci (Fig. 3 and S4)*.* According to the stringent screening conditions, only a small number of reasonable SNPs were associated with the shoot length, so the *P* value was reduced to < 10^− 5^ for subsequent analysis*.* In this way, a total of 10 intervals for biomass traits were identified in the control groups, while 29 intervals for biomass traits were present on all of the chromosomes other than chromosome 3 in the heat treatment group (Table S3)*.* For the dry weight and fresh weight of the treatment groups, the consistent intervals, *qDW_ind8* (3.09 Mb–3.29 Mb) and *qFW_ind8* (3.09 Mb–3.29 Mb), were identified on chromosome 8. Therefore, there are important candidate genes controlling each biomass traits under high temperature stress in this interval*.* It can be inferred that the expression of one or more genes in this interval is in response to high temperature stress by adjusting the dry weight and fresh weight characters of rice seedlings.

**Fig. 4:**
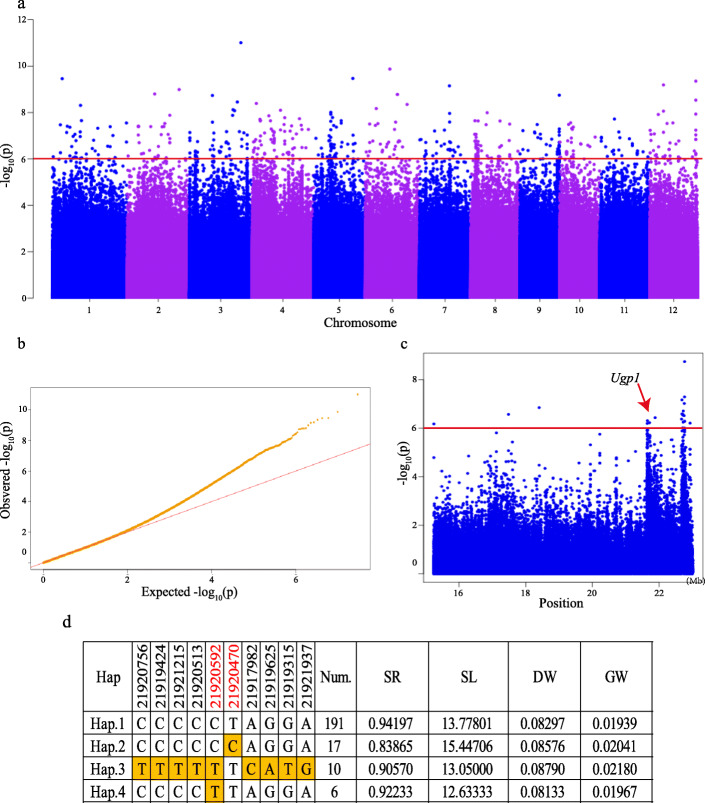
Correlation analysis of the survival rate of the natural rice population and analysis of known gene haplotypes. **a** Manhattan plot of survival rate (SR). **b** QQplot of survival rate. **c** Scatter plot of *Ugp1*. The arrow indicates that the site is the approximate site of *Ugp1*. **d** Gene structures (left) and biomass traits of different haplotypes (right) of *Ugp1*. Red colored numbers indicate the key SNPs among major haplotypes

There are 77 intervals containing 283 SNPs for survival rate after heat treatment (Fig. 4a, Table S3). Among them, *qSR_ind9–3* on chromosome 9 contains gene *Ugp1*, a known heat-tolerant gene at the heading stage [24] (Fig. 4c). As *Ugp1* is expressed during the whole growth and development period of rice [24], it is suggested that this gene could also have an effect on the rice seedling response to heat stress. To further determine the natural variation of *Ugp1*, we performed haploid genotype analysis using all of the non-synonymous SNPs in the coding region of this gene (Fig. 4d). A total of 10 SNPs in the coding sequence (CDS) region were detected, and four major haploid genotype were identified based on the 10 SNPs. Hap.1 is the main haploid genotype in nature population, and the varieties belonging to Hap.1 have higher survival rate after high temperature stress treatment in rice seedling stage. It can be found that the expression of *Ugp1* does have an effect on the response of rice seedlings to high temperature stress. Among 255 materials, a total of 191 varieties belong to the Hap.1 haploid genotype, which proves that Hap.1 has a higher utilization rate in the population. Compared with Hap.1, the survival rate of Hap.2 after the change of chr9_21920470 was low, but the biomass increased significantly. For Hap.3, all sites except chr9_21920470 changed. Although the survival rate of Hap.3 is also lower than that of Hap.1, the decline rate of survival rate is lower than that of Hap.2. Therefore, we judge that the chr9_21920470 locus has a stronger effect on the survival rate of rice seedlings than other loci. In Hap.4, where chr9_21920592 changes, compared with Hap.1, shoot length is lower. From this, we judge that this site may be related to plant growth.

### Compare results from linkage analysis and GWAS

**Fig. 5:**
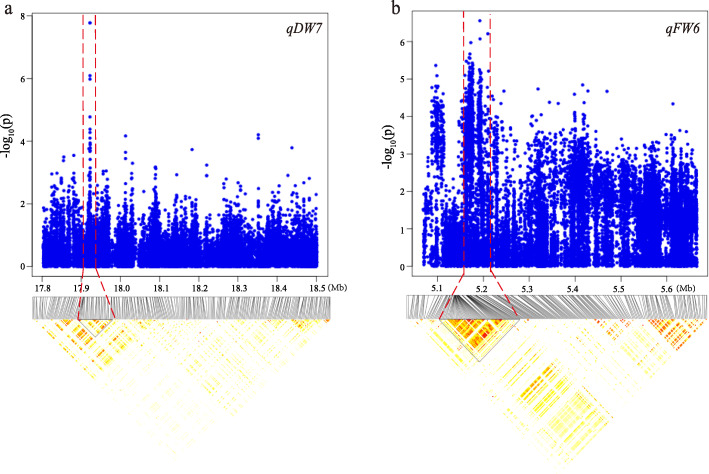
Manhattan plot (top) and LD heatmap (bottom) surrounding the peak of the candidate interval. Interval of linkage analysis and genome-wide association analysis in **a** *qDW7*. **b** *qFW6*. Dashed lines indicate the candidate region of the peak

We compared the QTLs obtained by linkage analysis with the candidate intervals identified by GWAS. Two groups of candidate regions identified on chromosome 7 and chromosome 6 were co-located by linkage analysis and association analysis. There was a 200 kb (5.59 Mb–5.70 Mb) coincidence interval in *qFW6* and *qFW_ind6* for the fresh weight trait in the treatment group. Analyzing the LD attenuation distance of this interval (Fig. 5), we further reduced the candidate interval to 69 kb (5.62 Mb–5.69 Mb). According to MSU V7.0 [25] annotated information, there are 13 candidate genes in this interval, including 12 genes involved in expression (Table S4). The same analysis was performed on the interval of 200 kb (17.82 Mb–18.02 Mb) in *qDW7* and *qDW_ind7* on chromosome 7, we reduced the candidate interval on chromosome 7 to 57 kb (17.90 Mb–17.95 Mb), and identified 6 genes (Table S4). To further investigate candidate genes, we performed homologous analysis on the obtained genes. The results of homologous analysis showed that the homologous gene *LECRK-VII.2* of *LOC_Os06g10790* was involved in the response to high temperature and drought in *Arabidopsis* [26]. The homologous gene *UGT73B3* [27] of *LOC_Os06g10860* and the homologous gene *UGT85A2* [28] of *LOC_Os07g30330* are involved in the stress response of *Arabidopsis*. The homologous gene *PHT3* [29] of *LOC_Os06g10810* is related to the oxidation-reduction reaction of *Arabidopsis thaliana*. These homologous genes on the one hand confirm the accuracy of the experimental results, on the other hand, further narrow the range of candidate genes in this study.

### Transcriptome analysis by RNA sequencing

**Fig. 6:**
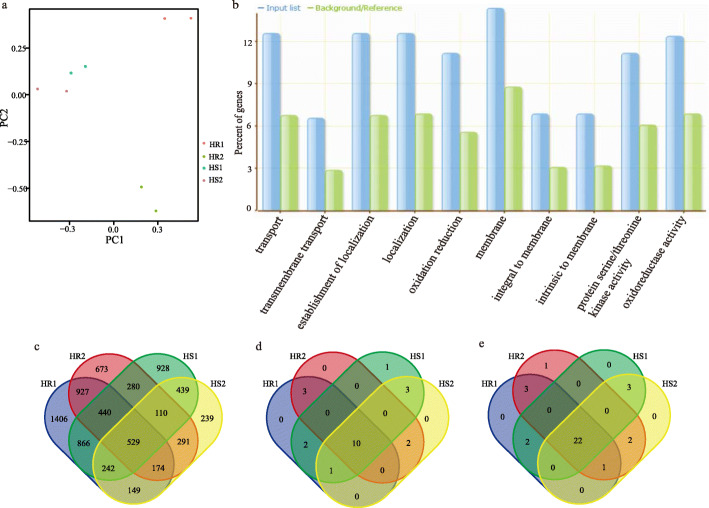
Genome-wide transcriptome analysis of heat stress between heat-tolerant and heat-sensitive rice varieties. **a** The PCA of Hierarchical clustering of all the samples after normalisation. **b** GO enrichment analysis of differential genes. The threshold of FDR < 0.05 was selected to identify significant enriched GO terms. **c** Venn diagrams showing the differential genes in response to heat stress. **d** genes up-regulated by redox-related pathways. **e** Redox-related pathways down-regulate genes. HR1 and HR2 are varieties with extremely high survival rate. HS1 and HS2 are varieties with extremely low survival rate

In order to study the transcriptome response of *indica* rice to high temperature in the seedling stage, two heat-resistant (HR) varieties and two heat-sensitive (HS) varieties were selected for RNA_seq analysis. The PCA results showed a difference between the heat-resistant group and the heat-sensitive group (Fig. 6a). After comparing the gene expression between the heat-resistant groups and the heat-sensitive groups, it was determined that a total of 529 genes were differential expression in the four groups (Fig. 6c), and it was considered that there were key genes responding to high temperature stress in rice seedling. Kyoto encyclopedia of genes and genomes (KEGG) analysis was performed on 529 differential expressed genes (DEGs). The results showed that 75 DEGs were significantly enriched in pathways such as metabolic pathways, pyruvate metabolism, starch and sucrose metabolism (Table S5). *OsCML4* [30], which is enriched in the mitogen-activated protein kinase (MAPK) signaling pathway-plant pathway, has been shown to improve the rice tolerance by removing reactive oxygen species and inducing other stress related genes in a form independent of ABA survival rate. This indicates that there are genes in DEGs genes that can respond to high temperature stress for subsequent research. Further, we identified *LOC_Os01g09450*, *LOC_Os03g59040* and *LOC_Os12g42980* in the GWAS candidated intervals *qSR_ind1–1, qSR_ind3–9*, and *qSR_ind12–7*, respectively. The three genes were located in the KEGG pathway in plant hormone signal transduction, metabolic pathways, and cysteine and methionine metabolism pathways, and the expression of *LOC_Os03g59040* was confirmed to be related to the stomatal conductance of rice [31]. We conclude that *LOC_Os01g09450*, *LOC_Os03g59040* and *LOC_Os12g42980* could be used as candidate genes for rice seedlings in response to high temperature stress. Based on the gene ontology (GO) enrichment analysis, most genes were enriched in the oxidation reduction, oxidoreductase activity, transmembrane transport and others (Fig. 6b). We focused on the 44 genes enriched in oxidation reduction and oxidation activity activities. Among the 44 genes identified, there are 10 down-regulated genes and 22 up-regulated genes (Fig. 6d, f). After a comparison with these genes and the intervals obtained in GWAS, it was found that *LOC_Os02g12890* was located on the *qSR_ind2–1* for survival rate. It was suggested that *LOC_Os02g12890* could be the candidate gene that may respond to high temperature stress in the rice seedling stage and directly affect the heat tolerance of rice seedlings.

**Fig. 7:**
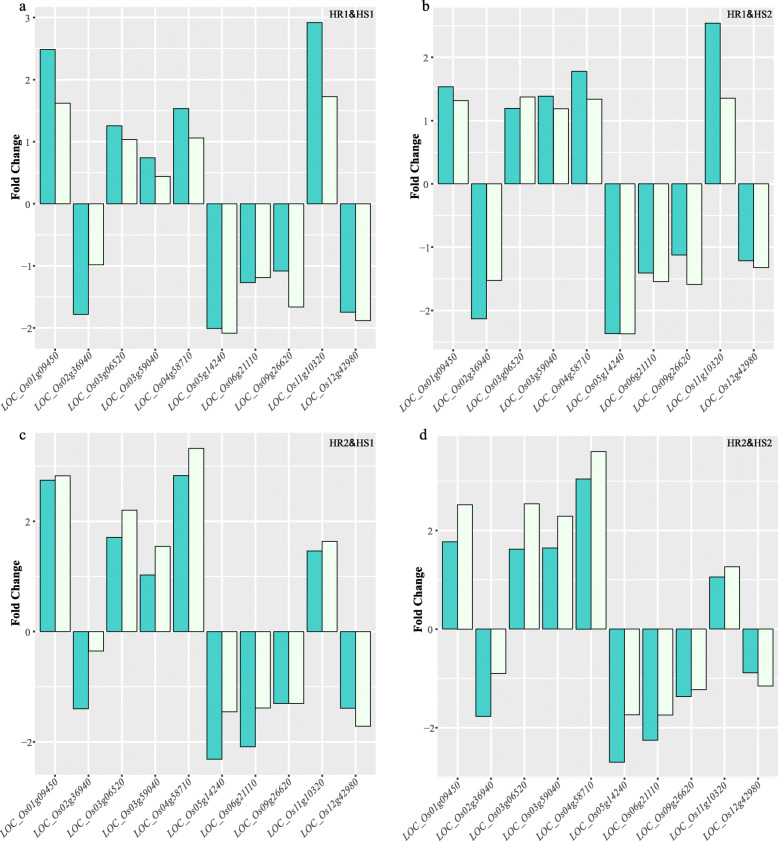
Validation of RNA-seq with qRT-PCR. **a** The figure shows the multiple relationship of the gene expression of the two groups of extreme materials after heat treatment, including the transcriptome FPKM and qRT-PCR quantitative results. **a** shows the processing of HR1 and HS1. **b** HR1 and HS2. **c** HR2 and HS1. **d** HR2 and HS2. HR1 and HR2 are varieties with extremely high survival rate; .HS1 and HS2 are varieties with extremely low survival rate

Based on the transcriptome data and the enriched results, we randomly selected 10 genes for quantitative real-time PCR (qRT-PCR) detection. In the differential gene analysis of different groups, the fragment per kilobase million (FPKM) and qRT-PCR results of each of the 10 genes have the same trend (Fig. 7 and S5), which proves that the transcriptome sequencing results are accurate.

In summary, a total of 23 candidate genes were co-localized by two or more methods, including 19 genes co-located by linkage analysis and GWAS and genes in 4 related pathways by GWAS and transcriptome analysis. 8 genes, such as *LOC_Os06g10790*, *LOC_Os06g10860*, *LOC_Os07g30330*, *LOC_Os06g10810*, *LOC_Os01g09450*, *LOC_Os03g59040*, *LOC_Os12g42980*, *LOC_Os02g12890*, etc. have been reported to be related to the plant response to heat stress due to homologous analysis or the focus has been reported in the follow-up research on the plant response to heat stress.

## Discussion

### The combination of linkage, association analysis and transcriptome provides effective information for target genes mining

Linkage analysis based on RIL and association analysis based on a natural population are two complementary approaches to revealing the candidate genetic variation leading to traits of interest. GWAS analysis can evaluate the diversified effect of many alleles with higher mapping resolution, which can make up for the inferiority of QTL analysis that the number of natural diversity is limited. Researchers have used linkage analysis or association analysis to identify heat-tolerant genes in rice previously [9–12, 32–34]. In other agronomic traits, it has also been confirmed that the combined application of linkage and association analysis can obtain more accurate candidate intervals [18]. In our study, a total of 116 loci were detected by GWAS, and only 20 loci were detected by linkage analysis. In the present study, two co-localized regions, a 200 kb interval in *qFW6* and *qFW_ind6,* and another 200 kb interval in *qDW7* and *qDW_ind7*, were detected by both linkage analysis and association analysis. These two reliable intervals should contain important candidate genes contributing to heat resistance in rice seedling stage. Further functional verification of these genes will reveal their underlying genetic and molecular mechanisms.

The transcriptome analysis is an effective approach for mining genes associated with given trait. In our study, we sequenced the transcriptome of two heat-tolerant varieties and two heat-sensitive varieties, compared the DEGs with GWAS, and obtained four candidate genes. These results suggest that the combination of linkage, association analysis and transcriptome is a powerful method for mining target genes responsible for rice heat resistance.

Therefore, in this experimental study, we not only used linkage and association analysis to narrow the candidate interval for this experiment, but also introduced and integrated the transcriptome analysis results of extreme performance groups. In addition to the 19 candidate intervals included in the two intervals co-localized by linkage analysis and association analysis, we also found 4 differential gene express enriched in related pathways.

### Exploration of genes related to high temperature in rice seedling

Abnormally high temperatures can affect the growth and development of rice*.* At present, temperatures in some areas have reached the critical value of the optimal temperature for rice growth, and rising temperatures would lead to a decline in rice production [35]. Therefore, exploring the genetic mechanism of heat resistance in rice is the key to cultivation of heat-resistant rice to adapt to the global warming environment. Research on the high temperature stress of rice was concentrated only on the late stage of rice growth and development, as an abnormally high temperature during reproductive growth had a direct impact on rice yield [6, 36, 37]. However, previous experiments have also proved that heat treatment affects rice seedling biomass, and high temperatures above 40 °C or long-term heat treatment affects the survival rate of rice [3, 4, 6, 7, 11, 38]. In particular, Li et al. completed the cloning of the heat-resistant gene *TT1* in 2015 [11], which greatly facilitated the breeding of heat-resistant and heat-resistant molecules in the later period. It is worth mentioning that the *TT1* gene was identified in wild African rice, and this gene was not identified in this experiment. The wild rice material analysis was not used in this experiment, so it was speculated that this is the reason that the gene was not found in this experiment. Nevertheless, the discovery of *TT1* did further confirms the feasibility and necessity of studying candidate genes that responded to high temperature stress at the seedling stage.

Experiments have shown that various biomass traits at the seedling stage can show different responses in different varieties of seedlings to high temperature. The three traits, SL, FW and DW, changed in the RIL and natural rice population after heat treatment (Fig. 1 and Table S1). At the same time, the calculation of heritability can also prove that the change of environmental temperature has an impact on rice seedlings.

After that, utilizing the different characteristics of the RIL population and the natural population, linkage analysis and association analysis were performed and obtained multiple candidate intervals. Among the 23 candidate genes we obtained that responded to high temperature in the seedling stage, it was also determined that the homologous genes of 8 genes were identified in *Arabidopsis thaliana* that were related to high temperature response. While this finding proves that the result is correct, it also confirms that high temperature does affect rice seedlings.

In addition, among these 8 genes, combining the results of FPKM and qRT-PCR (Fig. 7), it is not difficult to see that the three genes *LOC_Os01g09450*, *LOC_Os03g59040*, *LOC_Os12g42980* have obvious differences in response to high temperature stress. After experiencing high temperature in the seedling stage, *LOC_Os01g09450* and *LOC_Os03g59040* were significantly down-regulated in the expression of heat-sensitive varieties, while *LOC_Os12g42980* was up-regulated. The regulation mechanism of rice seedlings in response to high temperature is relatively complicated, and these related genes need to be studied and analyzed in depth.

### Identification of *Ugp1* favorable haploid genotype

In order to obtain the ideal high-temperature tolerance of rice, we also determined the favorable haploid genotype of the known gene *Ugp1* [24] identified in the GWAS results*.* By determining the survival rate of high-temperature stress seedlings among different haploid genotype, it was confirmed that the haploid genotype change had an impact on the survival rate of rice. The results of haploid genotype analysis indicated that the major haploid genotype consisting of non-synonymous SNPs in the coding sequence (CDS) within a single locus, represented the haploid genotype of most varieties*.* At the same time, the haploid genotype analysis of *Ugp1* [24] confirmed that haploid genotype analysis can identify key sites and favorable alleles of the target genes, providing a research direction for the identification of subsequent genes*.* This finding also facilitated the selection of optimal haploid genotype for subsequent breeding.

## Conclusions

In this study, three methods of linkage analysis, association analysis, and transcriptome analysis were used to complement and verify each other. New genes for rice seedlings responding to abnormally high temperatures were explored, and the genes identified by two or more methods were selected as candidate genes. Finally, based on homologous analysis and known gene information, it was determined that eight candidate genes could be focused on in subsequent studies. These results provide a reference for rice heat-resistant gene cloning and molecular breeding.

## Methods

### Plant materials

The rice variety 93–11, PA64s and their 124 RILs were used for linkage analysis [20]. A natural population of 255 Asian cultivated rice varieties with high genetic diversity from the 3000 rice project [23] was used for association analysis (Table S3). All materials are presented from International Rice Research Institute (IRRI) and Institute of Crop Sciences, Chinese Academy of Agricultural Sciences (ICS·CAAS).

### Seedling culture and high-temperature stress treatment

Intact seeds of the RIL and natural population were selected and immersed in deionized water for one night, and then transferred to a 32-well tray filled with nutrient solution. The nutrient solution was the conventional nutrient solution configuration of the International Rice Research Institute [39]. The pH value of the nutrient solution was adjusted between 5.5 and 6, and the nutrient solution was replaced once every three days. On the 15th day, the seedlings of the RIL and natural population required to be heat-treated were placed in an incubator at 45 °C for 52 h [10]. On the 20th day, the biomass traits, including plant height, dry weight, fresh weight, and seedling survival rate of RIL and natural population with or without heat stress treatment, were measured and recorded.

### Statistical analysis of data

We conducted a total of three repeated experiments. In each experiment, five plants from each material were randomly selected for statistics. The phenotype data with or without seedling heat stress treatment were statistically analyzed by R × 64 3. 4. 2.

### Linkage analysis

Linkage analysis was performed according to a genetic map constructed in previous study [20] by combining genotypic analysis and phenotypic characterization of 124 RILs. The QTL of RIL was analyzed by composite interval mapping (CIM) [40] using Windows QTL Cartographer V2.5 software (http://statgen.ncsu.edu/qtlcarl/WQTLCart.htm,2008). The threshold of logarithm of odds was set as 2.5 for the presence of QTLs and physical locations of the obtained QTLs were determined according to a high-density SNP-labeled recombination bin map [20]. The detected QTLs were named by reference to the method proposed by McCouch [41].

### Association analysis

Association analysis of genotype and phenotyps were performed with the efficient mixed-model association eXpedited (EMMAx) [42]. The genotype data were referenced with sequencing information released for the 3 k rice database [23]. SNP site settings with a deletion rate greater than 40% and a minimum allele frequency (MAF) of less than 5% were filtered out. When the site had a significant level, *P* < 10^− 5^, it was considered to be associated with the trait and was used for subsequent analysis. The relevant drawings in the subsequent analysis were completed by the corresponding drawing package of R × 64 3. 4. 2. The method for determining and naming GWAS candidate intervals refers to the method [43].

### Transcriptome analysis

Two varieties were selected according to the highest and lowest survival rates of the natural population after heat treatment, respectively, and a total of four varieties were used for transcriptome analysis. The RNA of the leaves was extracted using TRIzol reagent according to the manufacturer’s guidelines, and two biological replicates were measured for each variety. In order to ensure that the RNA sequencing libraries had high-quality RNA, agarose gel electrophoresis, and spectrophotometry were used to check RNA concentration and purity. RNA of a total of 8 samples (4 varieties´ 2 biological replicates after heat treatment) were extracted for RNA sequencing (RNA-seq) library construction. Each library with insert sizes of 300-bp using paired end method was constructed using the TruSeq RNA Library Preparation Kit, version 2 (Illumina, USA) according to manufacturer’s guide. The libraries were sequenced using 150-bp paired-end Nova-PE150 sequencing platform. We generate 350,454,582 cleaned reads of 16 libraries ranging from 22.28 to 51.96 million per library (Table S6). TOPhat2 software [44] was used to align the cleanup data to the reference genome^[25]^ and gene expression was quantified by FPKM using the Cufflinks [44] default parameters. We use nipponbare (MSU V7.0 [25]) as the reference genome. The genes with adjusted *P* values less than e^− 5^ and log fold change (FC) absolute value higher than 0.58 were considered as differentially expressed genes (DEGs) [23]. Finally, Gene Ontology (GO) enrichment analysis was performed for DEGs using an online tool agriGO [45] and the GO terms were assigned to the GO categories biological process (BP), molecular function (MF), and cellular component (CC). The threshold of FDR < 0.05 was selected to identify significant enriched GO terms.

### qRT-PCR

Total RNA were extracted from leaves of treated seedlings using TRIzol Reagent (Invitrogen). After treatment with DNase I, approximately 2 μg of the total RNA were synthesized the first-strand cDNA using HiScript III RT SuperMix for qPCR (+gDNA wiper) (Vazyme) with oligo (dT) as a primer. qPCR reactions labeled by ChamQ Universal SYBR qPCR Master (Vazyme) were performed on a cycler apparatus (Bio-Rad CFX96). The data were analyzed by the 2-DCT method. Rice *Actin* was used as internal controls. The qPCR primers were listed in Table S7. Two biological replicates were included.

## Supplementary Information

**Additional file 1: Table S1.** Traits statics during the seedling stage of RIL, together with their parents and natural population. **Table S2.** 255 varieties included in the natural indica population [23]. **Table S3.** Candidate QTLs identified by GWAS*.* **Table S4.** Annotation information of candidate genes identified in GWAS and linkage analysis. **Table S5.** KEGG pathway analysis of DEGs. **Table S6.** Transcriptome sequencing data and mapping results. **Table S7.** The primer sequence of qRT-PCR.

**Additional file 2: Fig. S1.** Material response to heat stress.

**Additional file 3: Fig. S2.** Normal distribution maps of biomass traits and survival rate in the RIL in control and treatmet groups.

**Additional file 4: Fig*.*** **S3.** Normal distribution maps of biomass traits and survival rate in the natural population in control and treatment groups*. (PDF 385 kb)*

**Additional file 5: Fig. S4.** QQplots for Genome-wide association analysis in the treatment and control groups of the natural population.

**Additional file 6: Fig. S5.** Expression of 10 genes randomly selected from RNA-seq data.

## Data Availability

The datasets used during the current study are available from PRJNA610667.

## Abbreviations

BP: Biological process
CC: Cellular component
CDS: Coding sequence
CIM: Composite interval mapping
DEGs: Differential expressed genes
DW: Dry weight
EMMAX: Efficient mixed model association eXpedited
FC: Fold change
FPKM: Fragments per kilobase million
FW: Fresh weight
GO: Gene ontology
GWAS: Genome-wide association analysis
HS: Heat sensitive
HR: Heat resistant
ICS·CAAS: Institute of Crop Sciences, Chinese Academy of Agricultural Sciences
IRRI: International Rice Research Institute
KEGG: Kyoto encyclopedia of genes and genomes
LD: Linkage disequilibrium
LOD: Logarithm of odds
MAF: Minimum allele frequency
MAPK: Mitogen-activated protein kinase
MF: Molecular function
qRT-PCR: Quantitative real-time PCR
QTL: Quantitative trait locus
RNA_seq: Ribonucleic acid sequencing
RILs: Recombinant inbred lines
SL: Shoot length
SNP: Single nucleotide polymorphism
SR: Survival rate
