# Supplementary figures and images for: Linkage analysis, GWAS, transcriptome analysis to identify candidate genes for rice seedlings in response to high temperature stress
Source: BMC Plant Biol. 2021 Feb 9;21:85. doi: 10.1186/s12870-021-02857-2 (PMC7874481; doi:10.1186/s12870-021-02857-2)

a

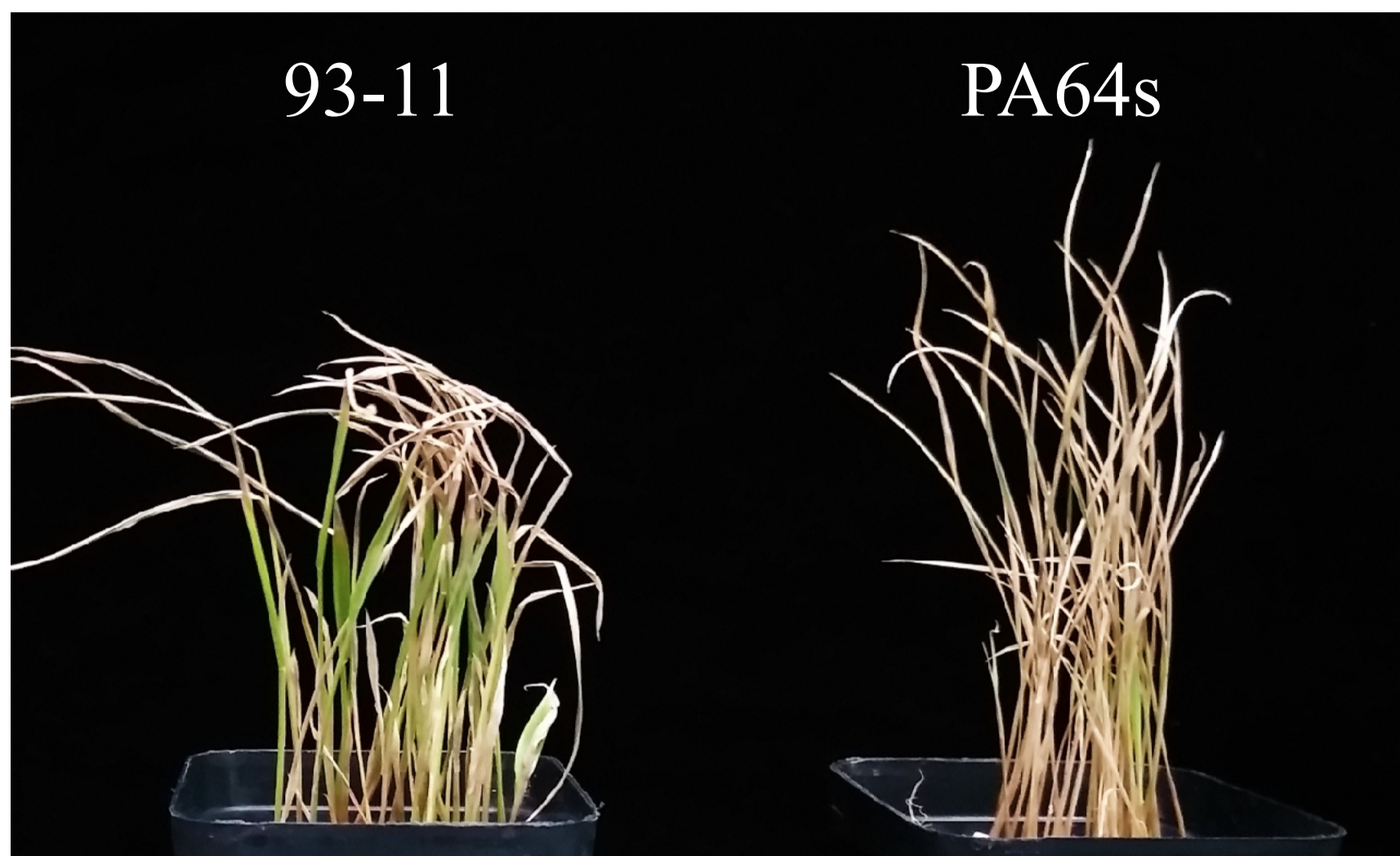

b

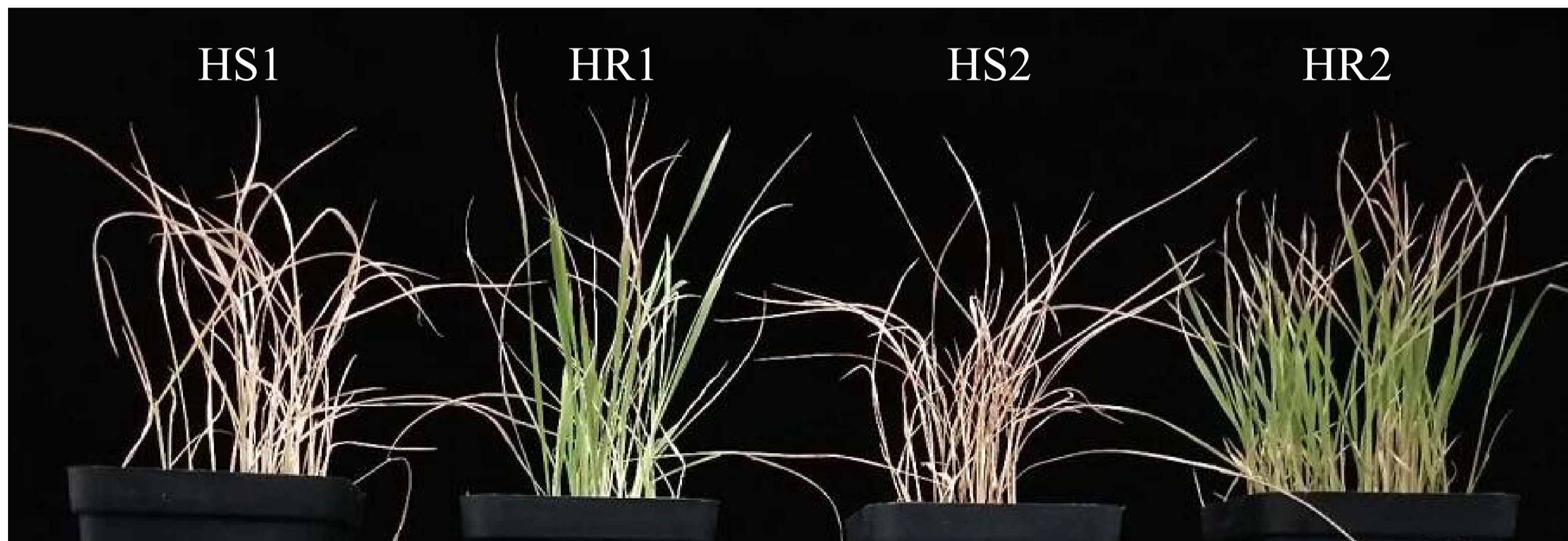

Supplement: Supplementary file 2 — Additional file 2: Fig. S1. Material response to heat stress. [file 12870_2021_2857_MOESM2_ESM.pdf]

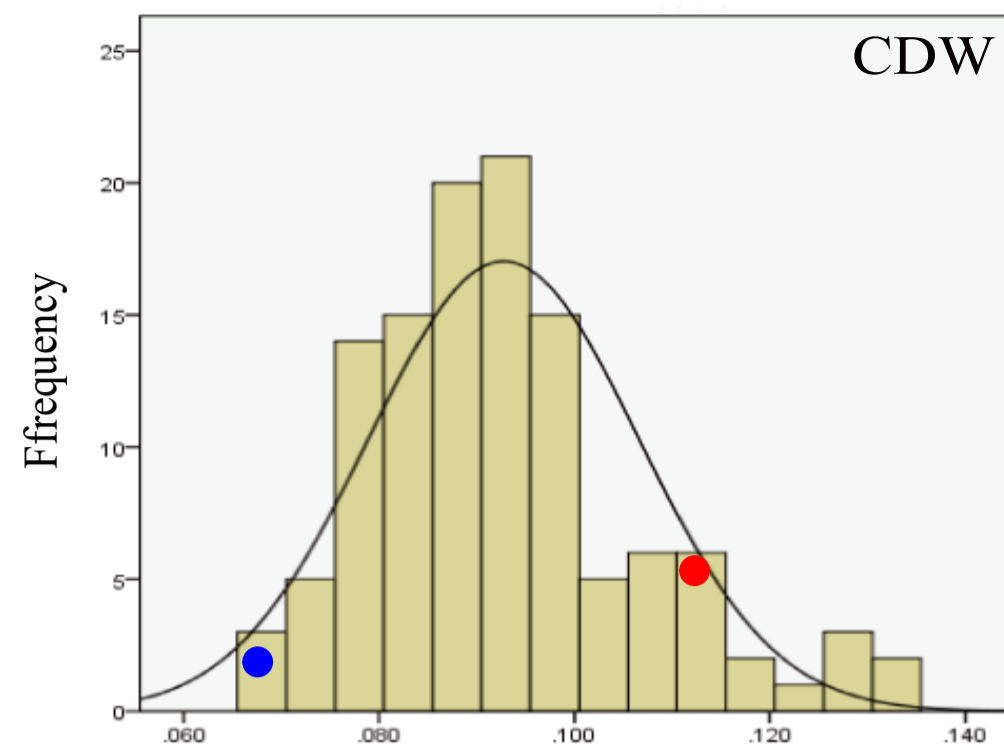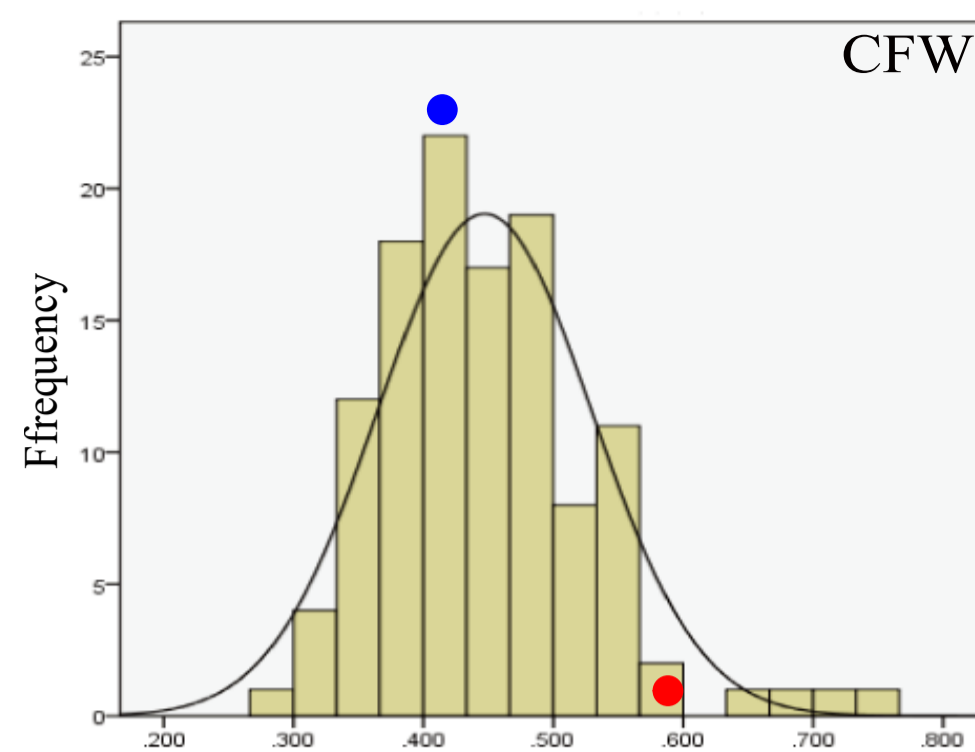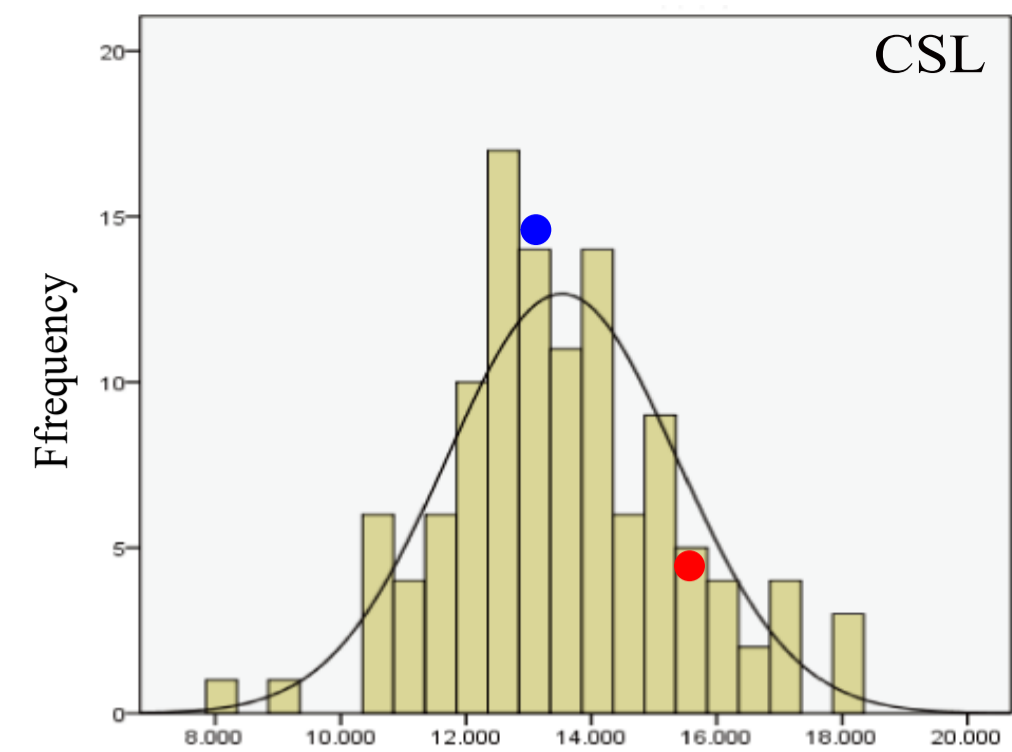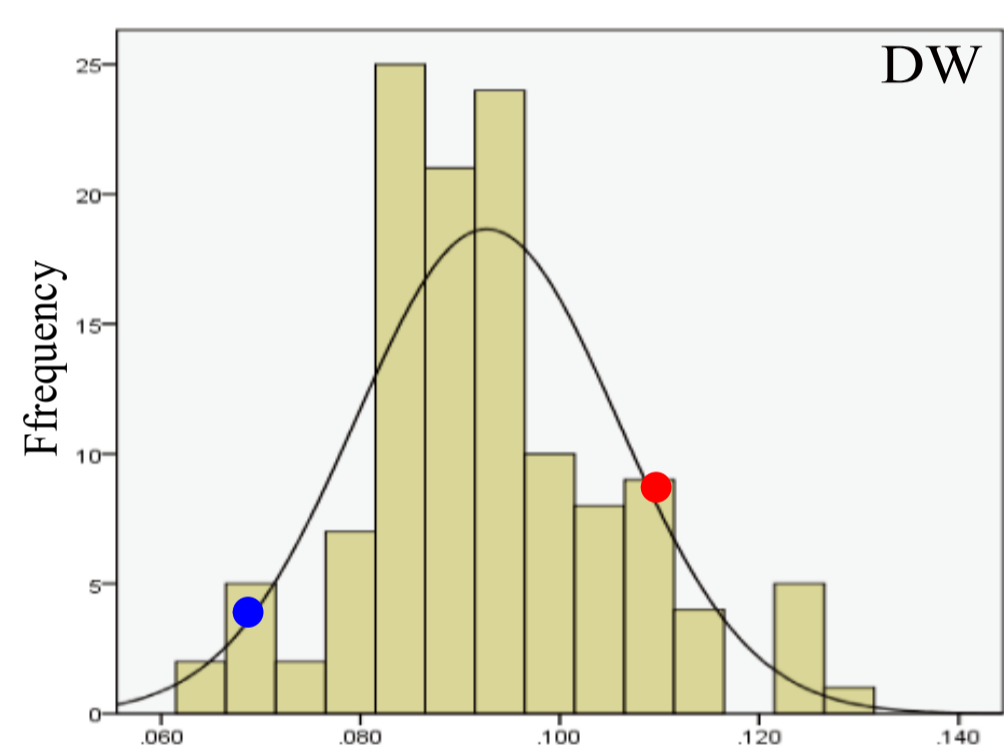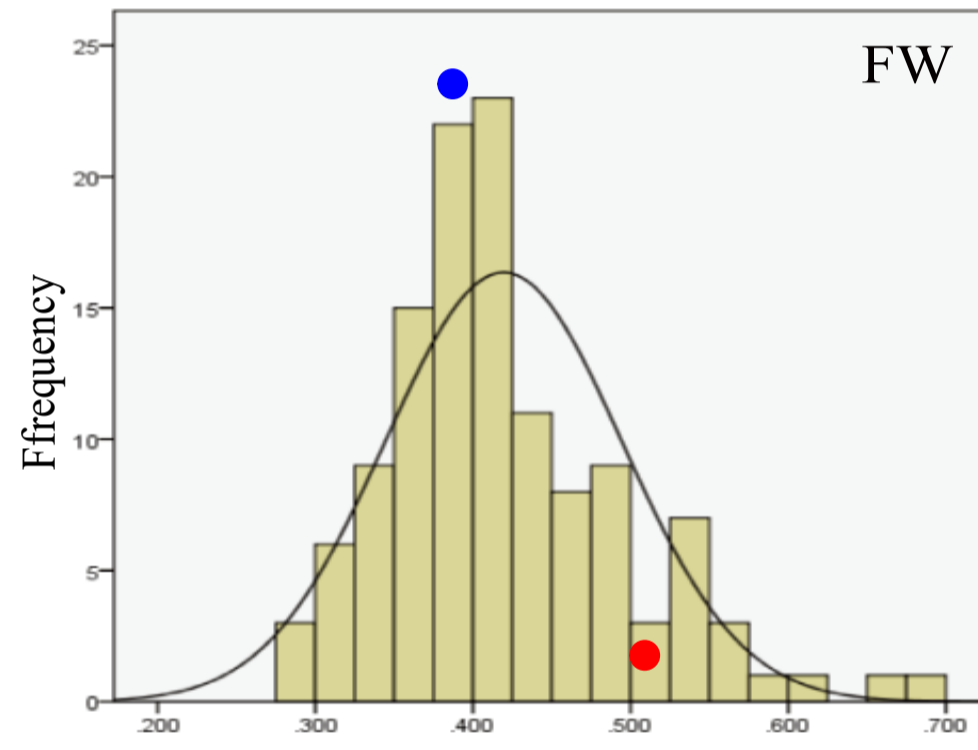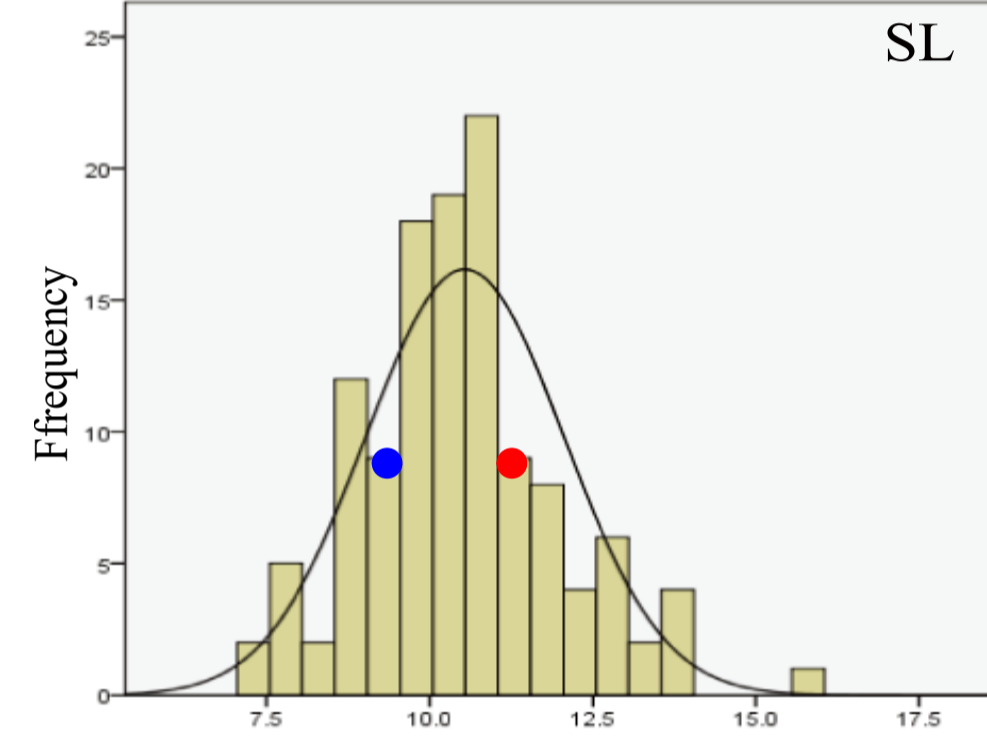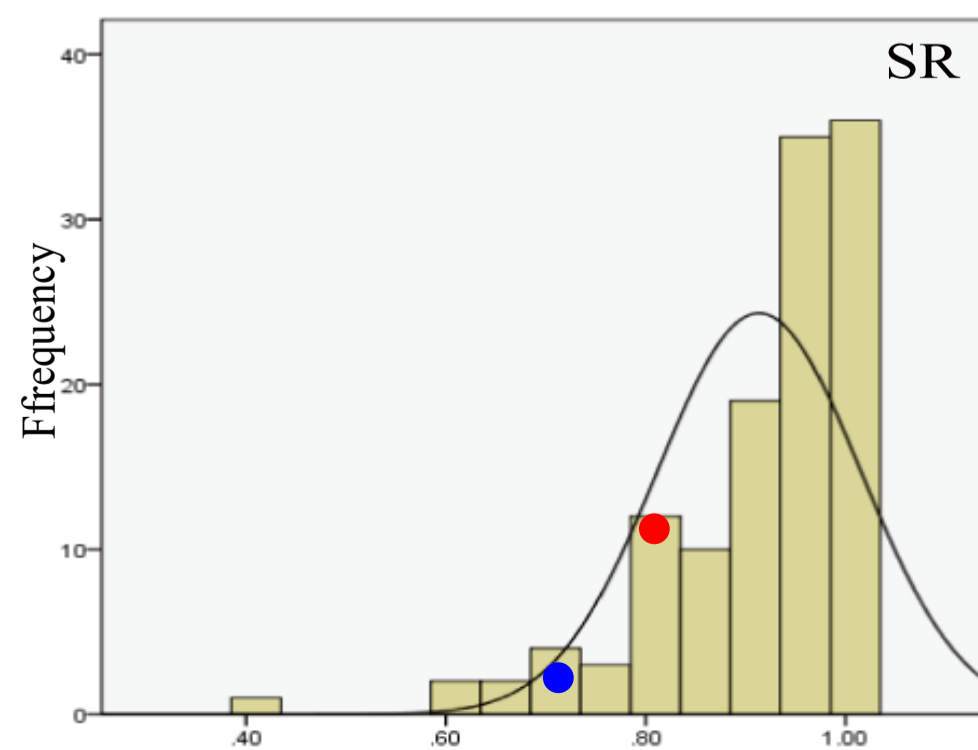

Supplement: Supplementary file 3 — Additional file 3: Fig. S2. Normal distribution maps of biomass traits and survival rate in the RIL in control and treatmet groups. [file 12870_2021_2857_MOESM3_ESM.pdf]

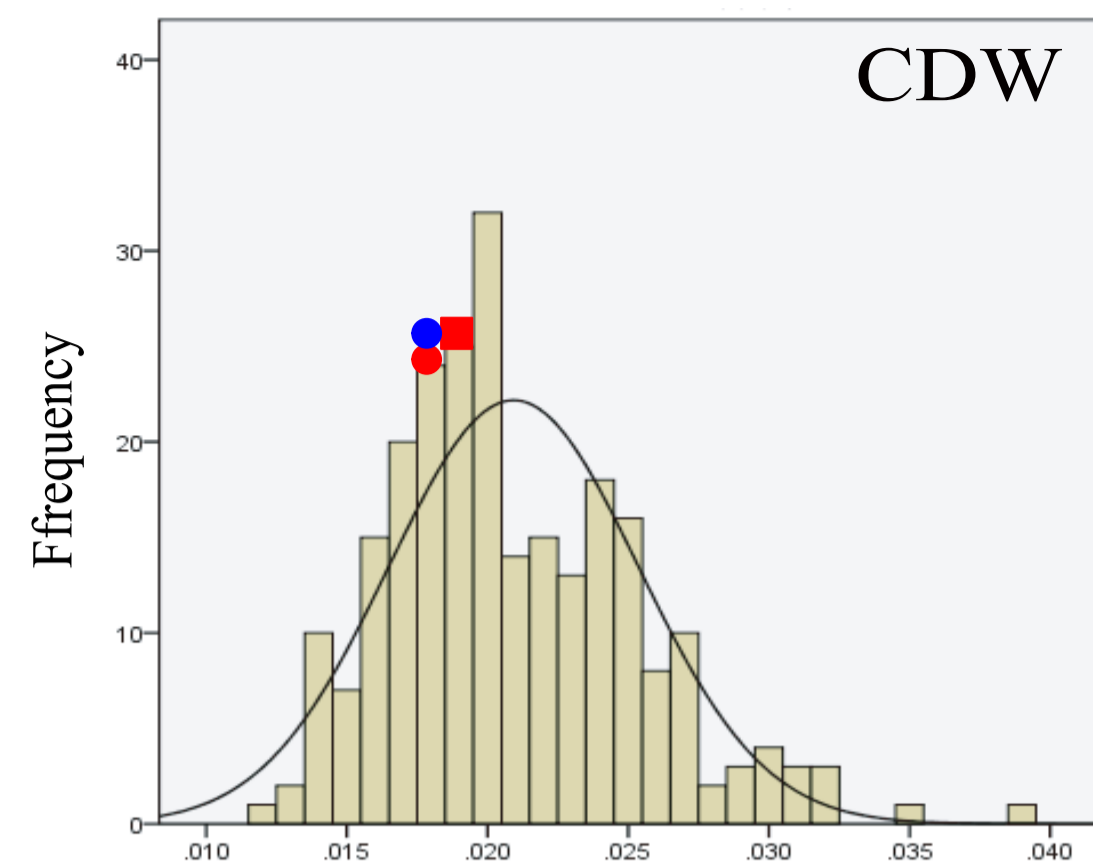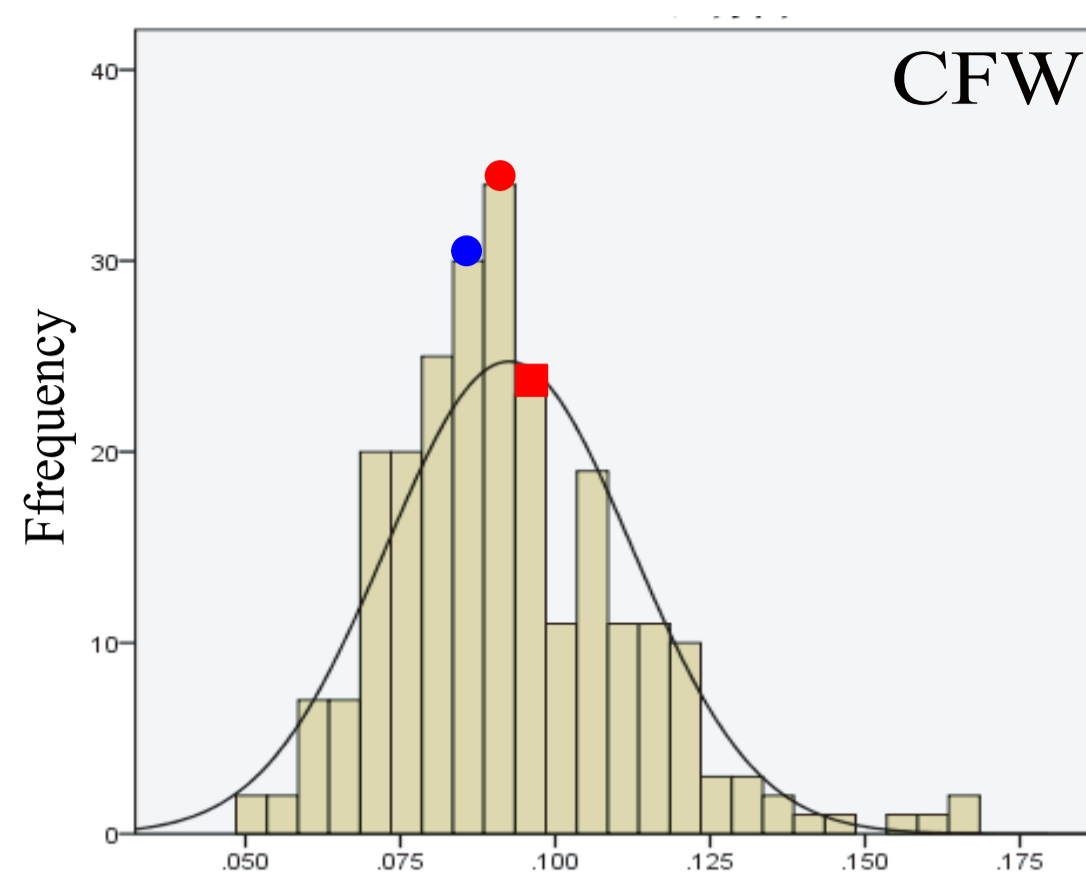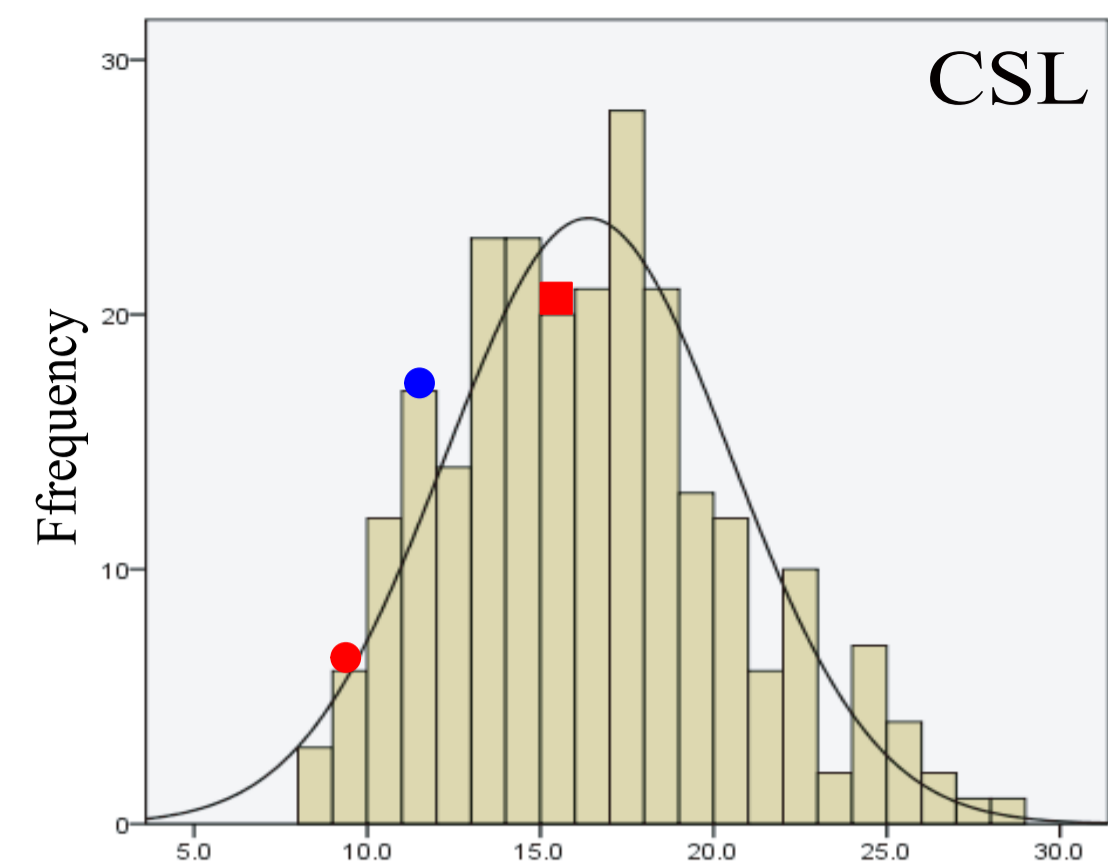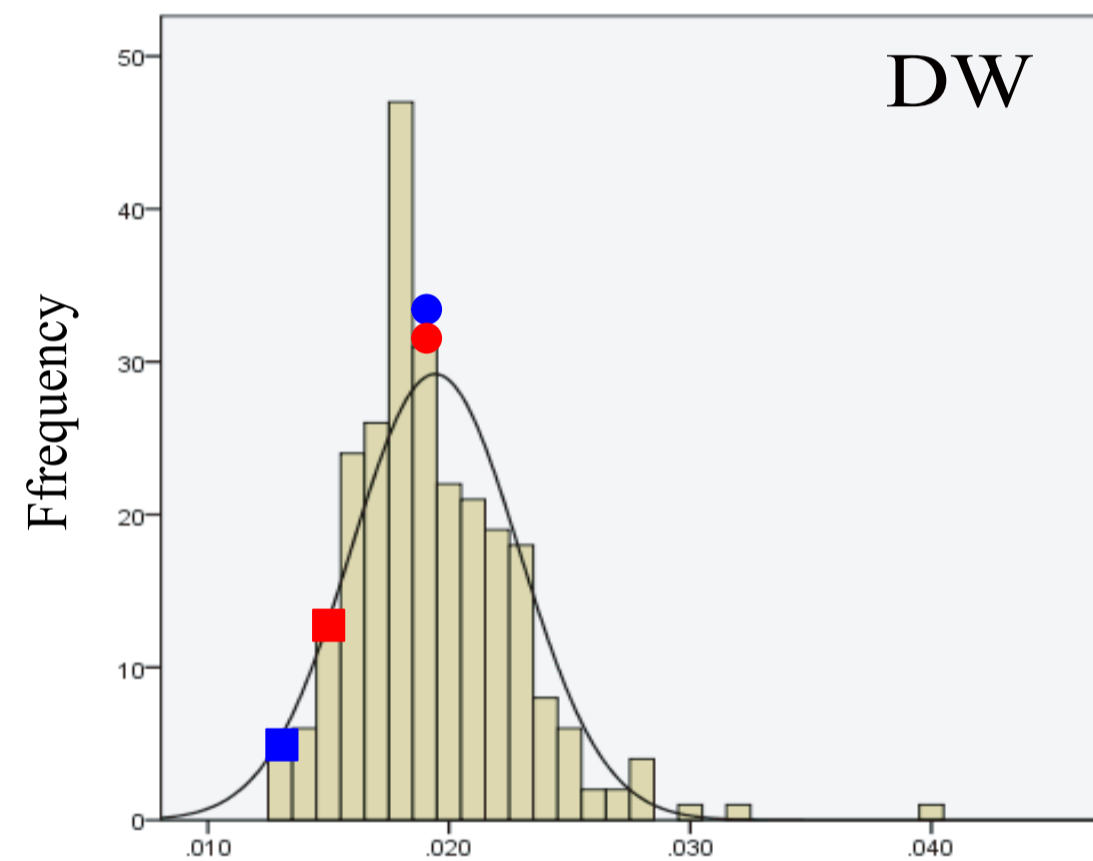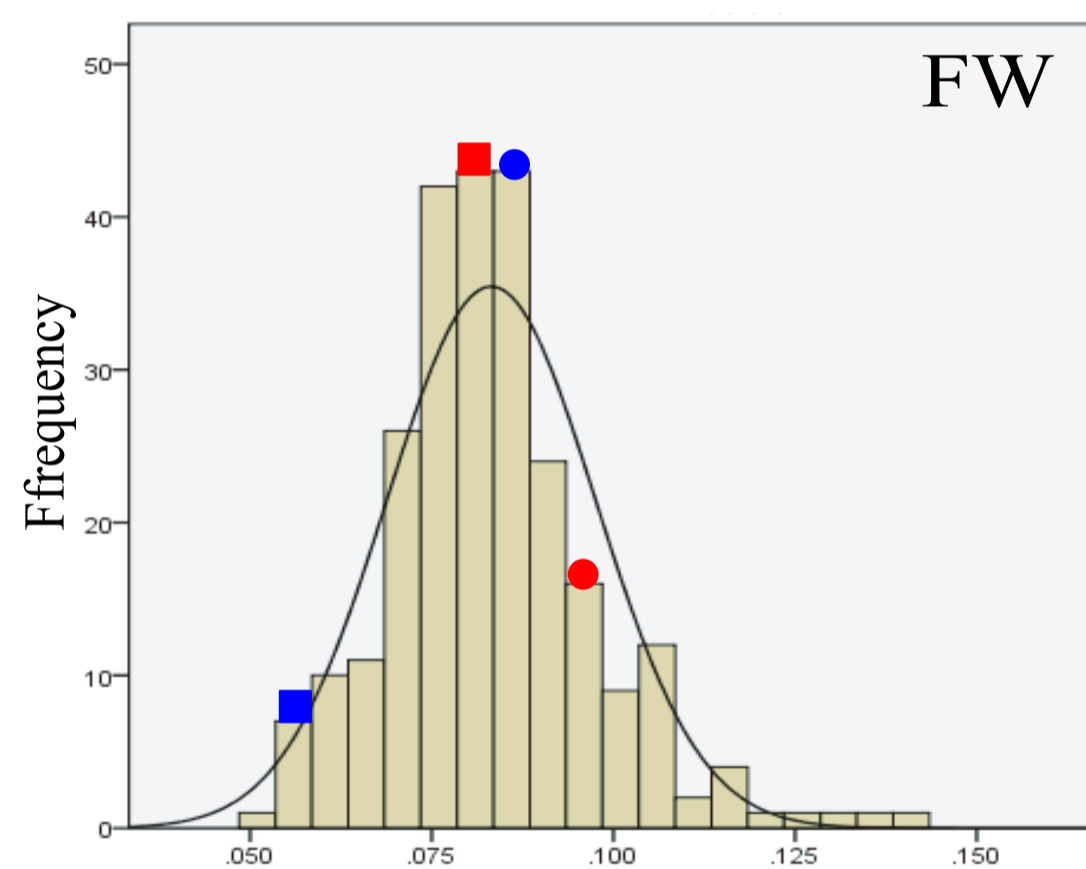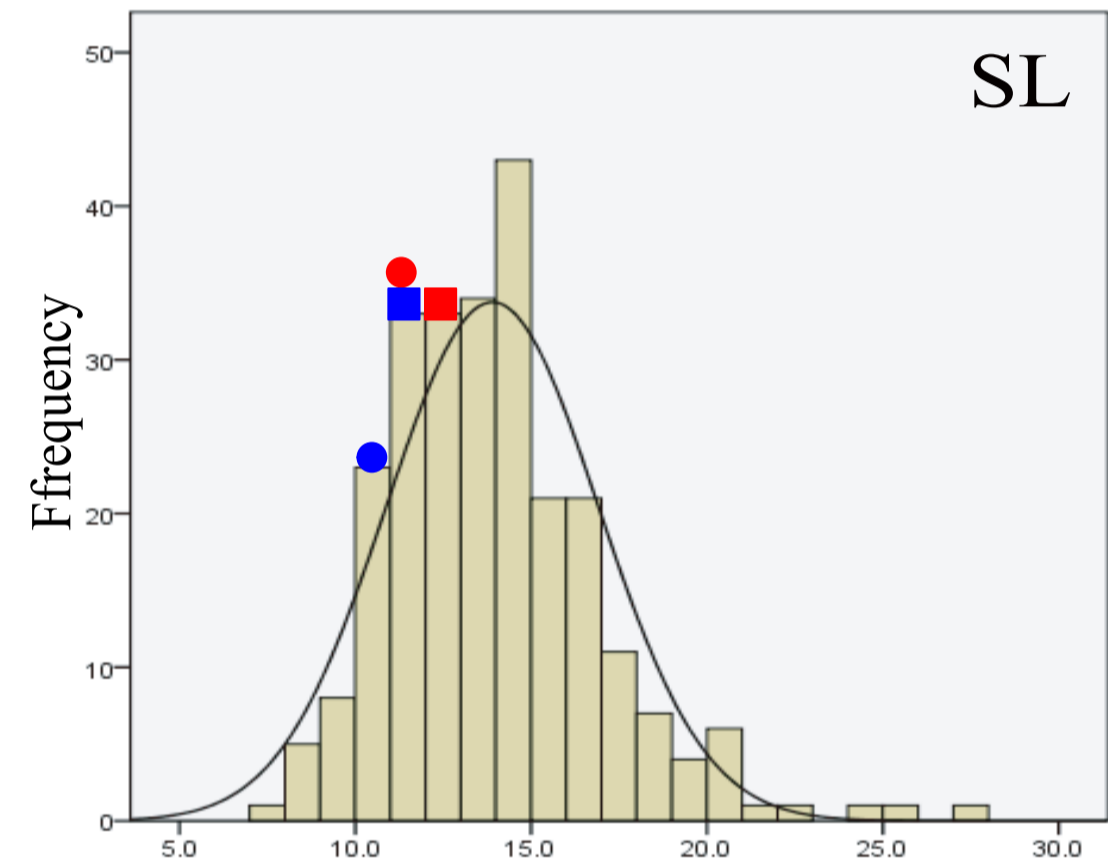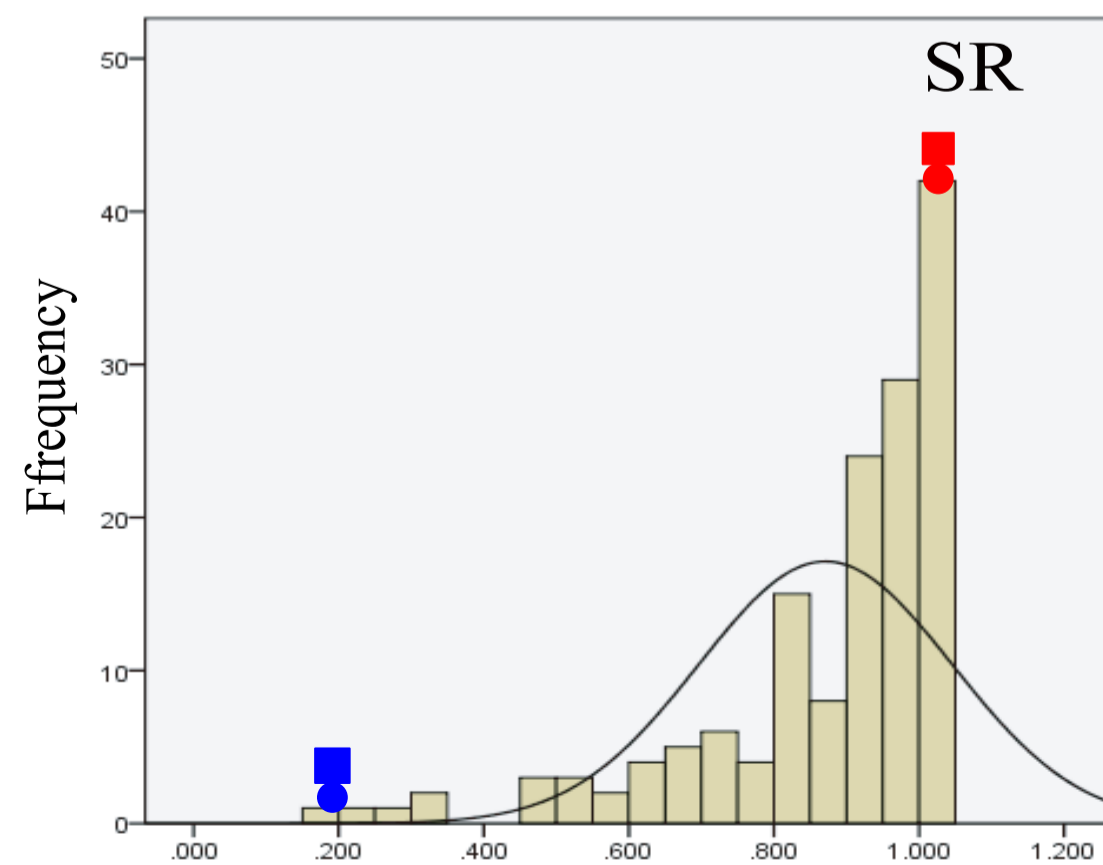

Supplement: Supplementary file 4 — Additional file 4: Fig. S3. Normal distribution maps of biomass traits and survival rate in the natural population in control and treatment groups. (PDF 385 kb) [file 12870_2021_2857_MOESM4_ESM.pdf]

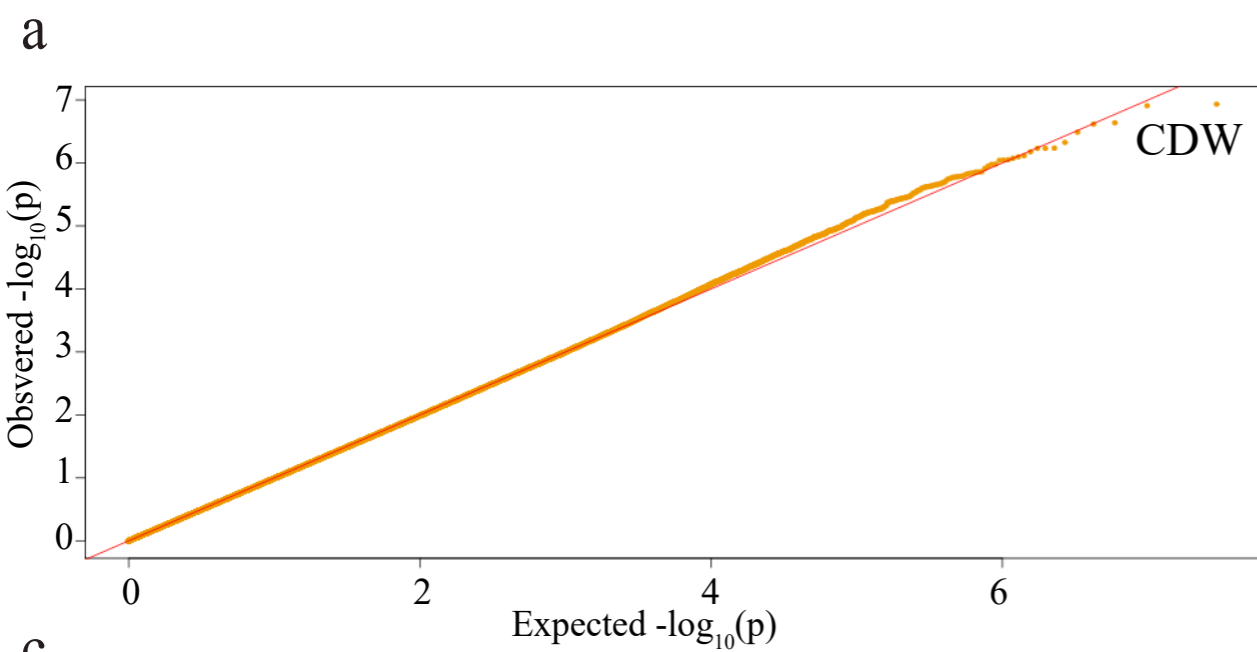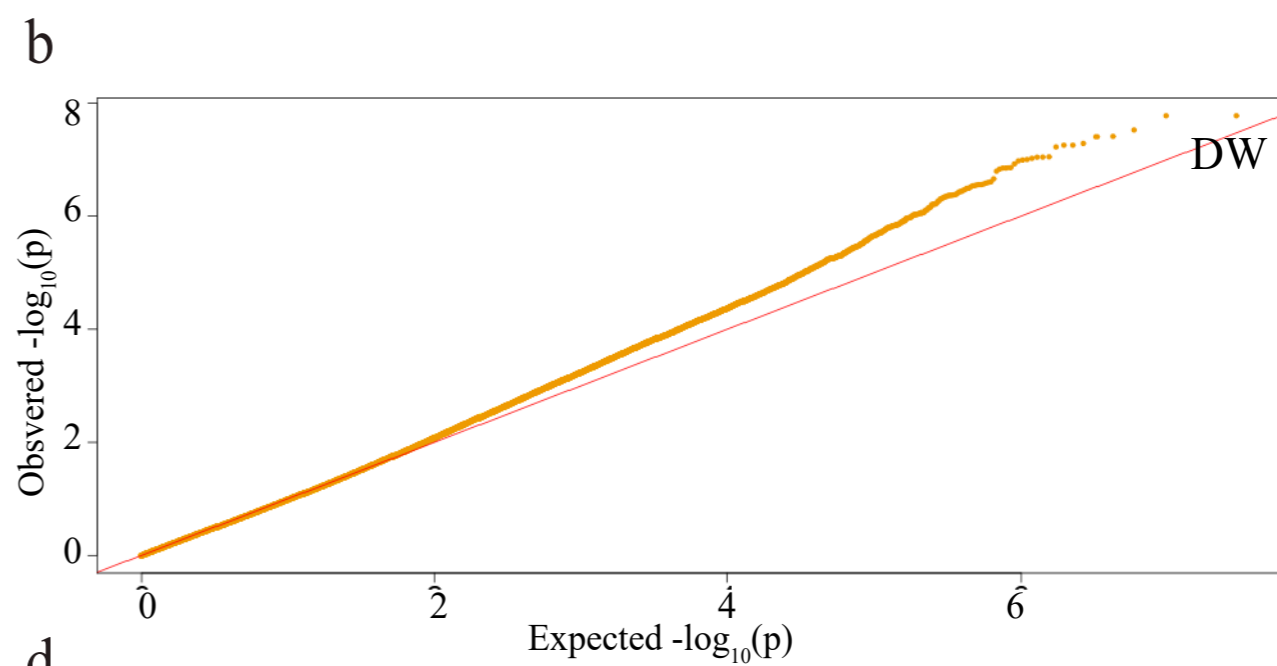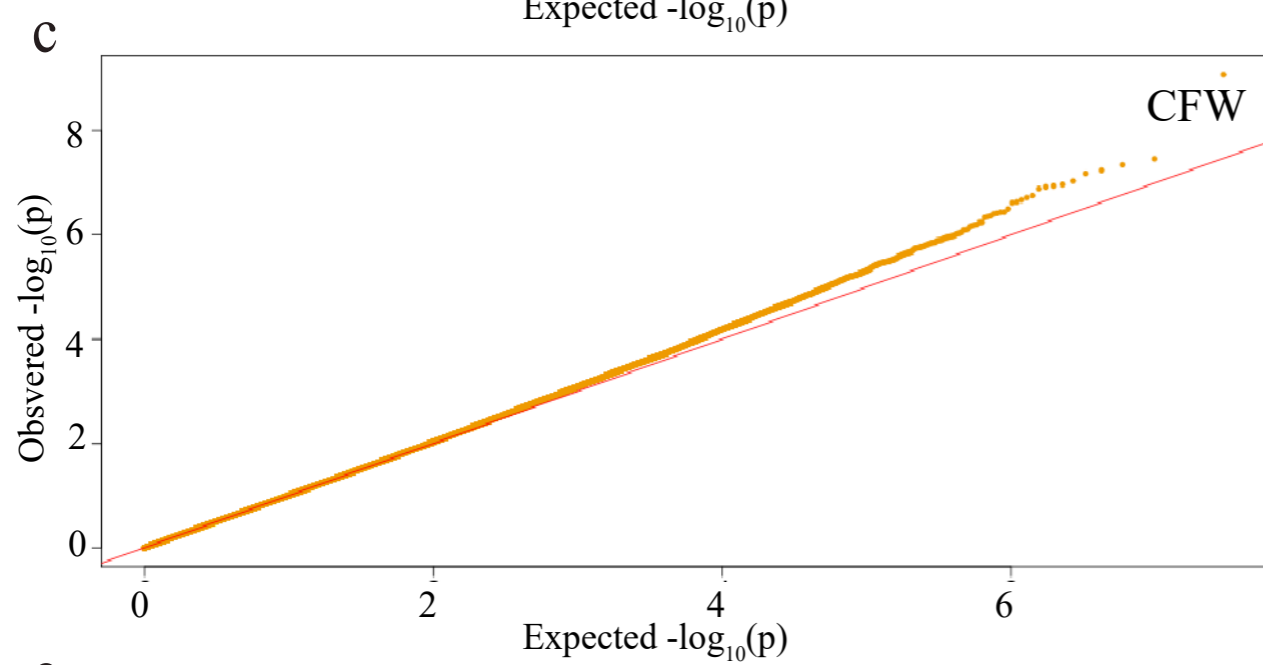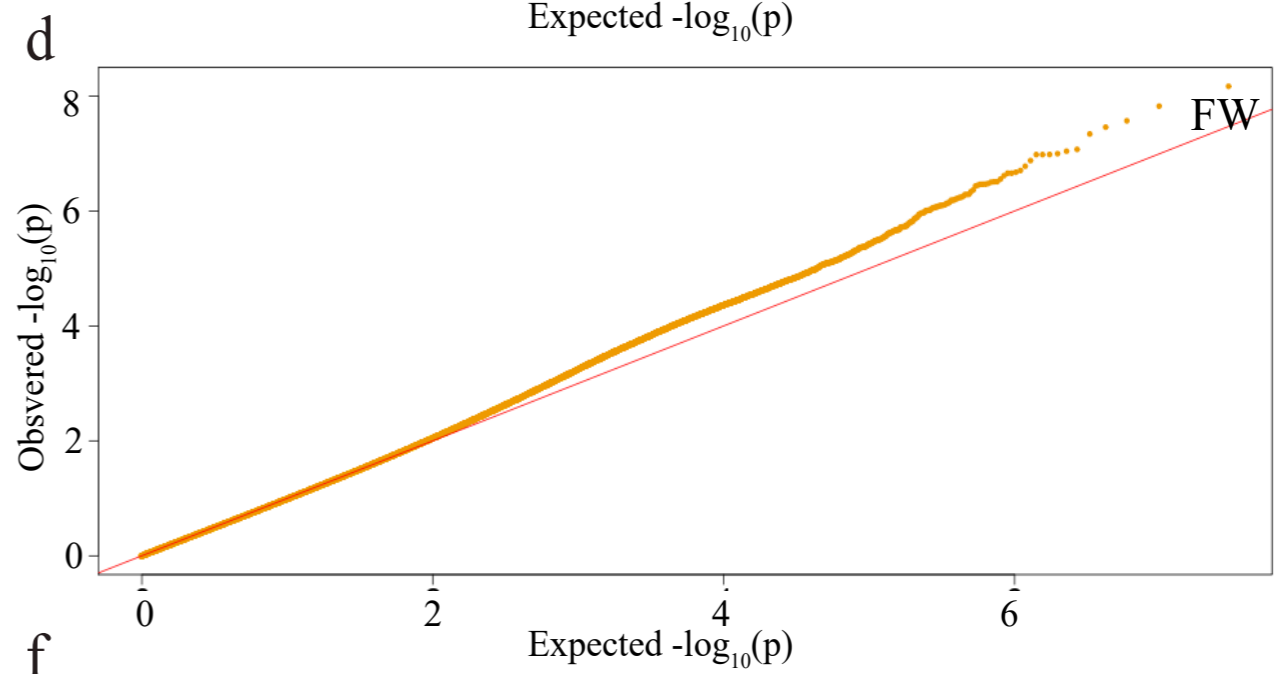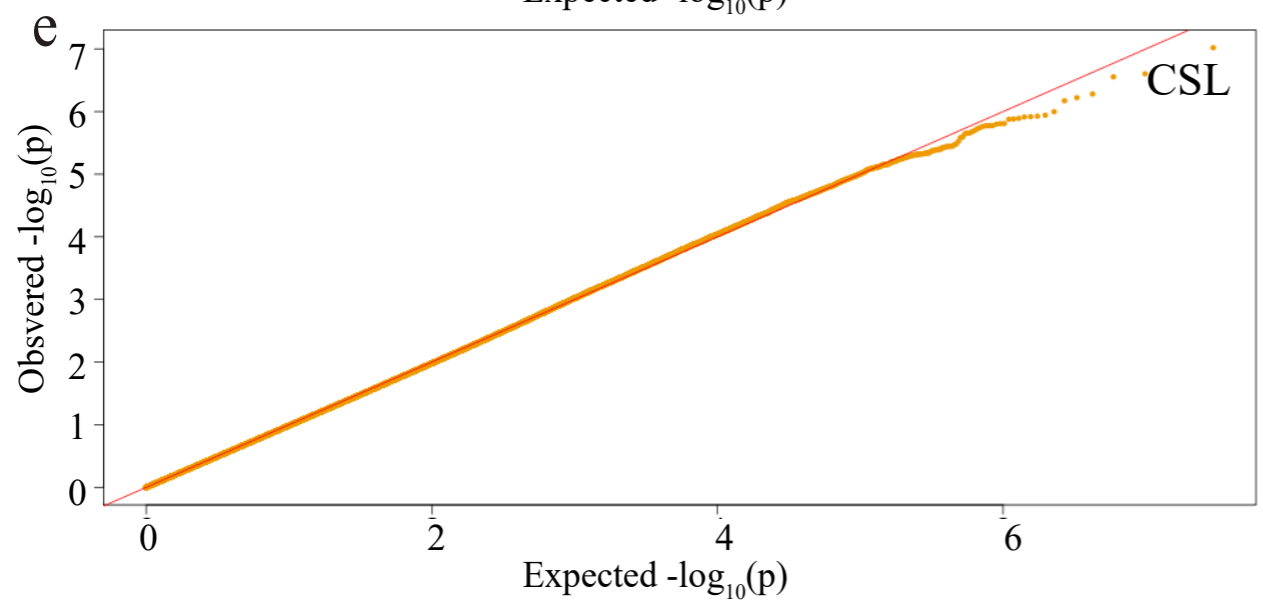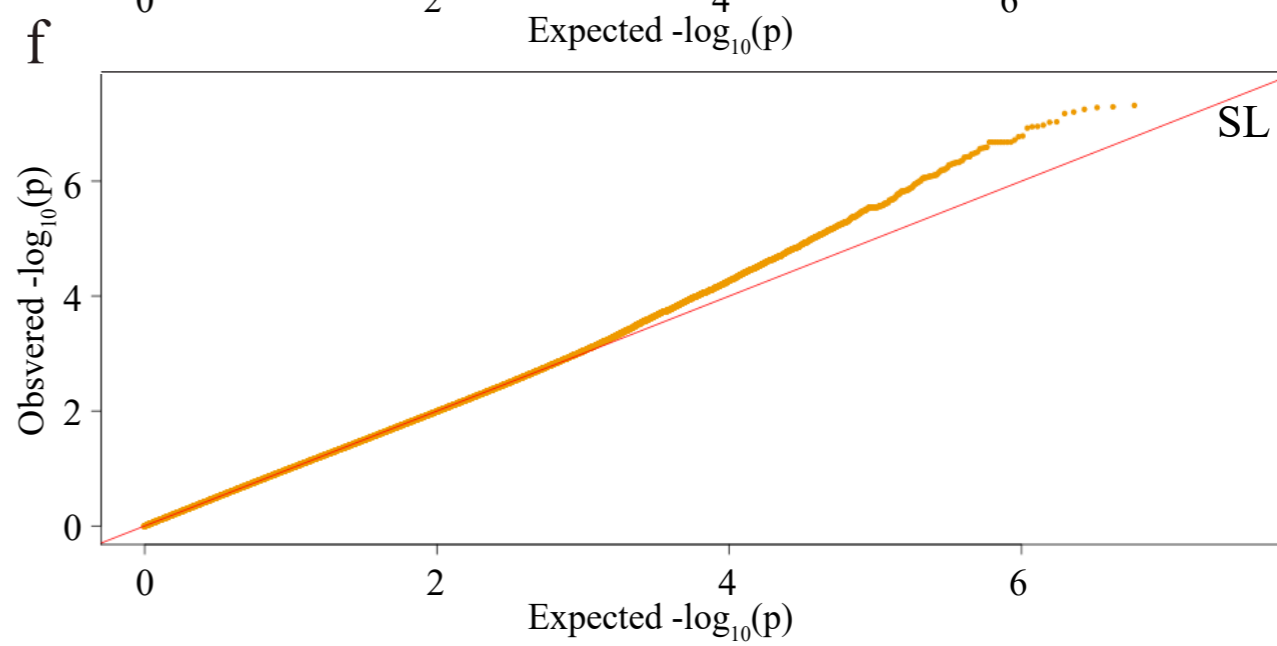

Supplement: Supplementary file 5 — Additional file 5: Fig. S4. QQplots for Genome-wide association analysis in the treatment and control groups of the natural population. [file 12870_2021_2857_MOESM5_ESM.pdf]

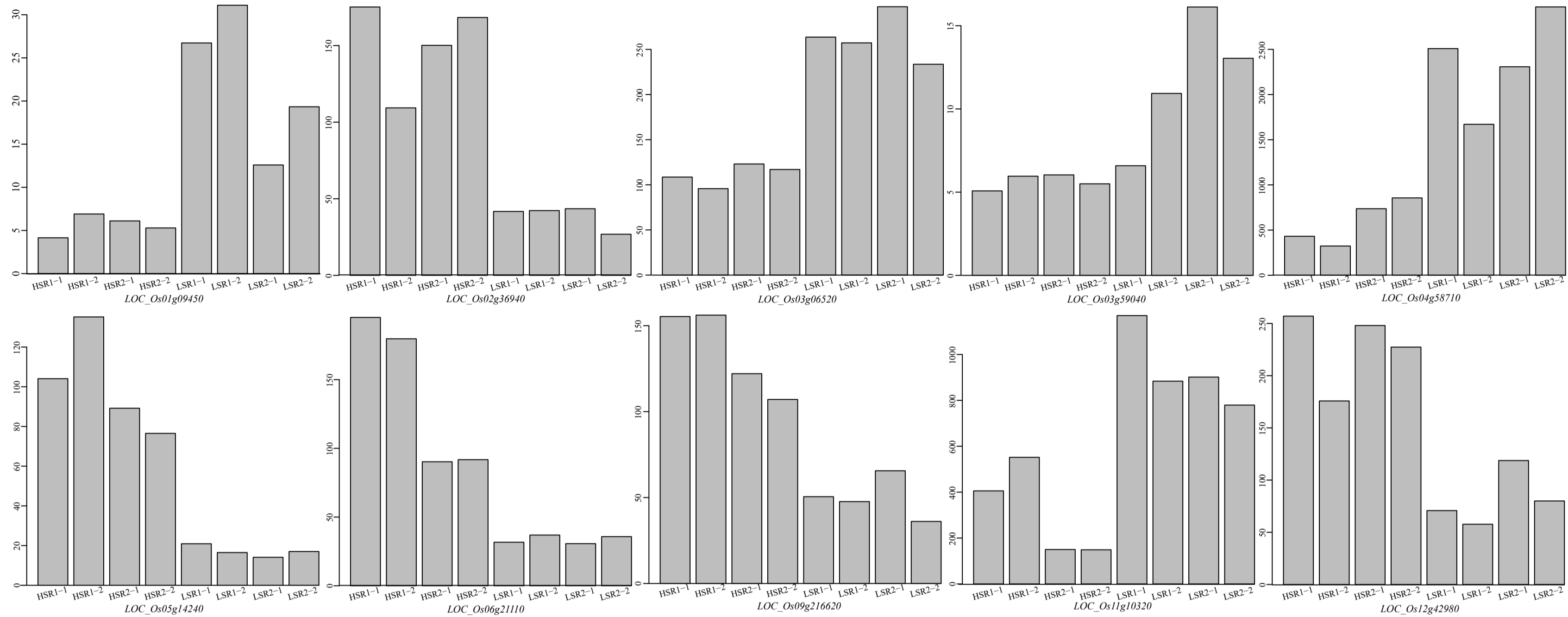

Supplement: Supplementary file 6 — Additional file 6: Fig. S5. Expression of 10 genes randomly selected from RNA-seq data. [file 12870_2021_2857_MOESM6_ESM.pdf]
